# Hepatic predominant presentation of Kawasaki disease in adolescence case report and review of literature

**DOI:** 10.1186/s12876-020-01461-2

**Published:** 2020-10-27

**Authors:** Krishan Pratap, Logan S. Gardner, David Gillis, Martin Newman, Dana Wainwright, Roger Prentice

**Affiliations:** 1 Department of Clinical Immunology and Allergy, Royal Brisbane and Women’s Hospital, Butterfield Street, Herston, Brisbane, QLD 4029 Australia; 2 School of Medicine, University of Queensland, Brisbane, Australia; 3 Greenslopes Private Hospital, Brisbane, Australia

**Keywords:** Kawasaki, Hepatitis, Liver, Adolescent, Case report

## Abstract

**Background:**

Kawasaki Disease (KD) is the most common paediatric vasculitis affecting small to medium arteries. Although the average age of diagnosis is 3.4 years with a well-defined clinical presentation, older patients with KD including adolescent and adult patients demonstrate a less classical presentation with prominent findings including hepatitis, cervical lymphadenopathy, and arthralgia. We describe a case of an adolescent presentation of Kawasaki Disease presenting with a predominantly cholestatic hepatic picture.

**Case presentation:**

We describe a case of KD in a 16-year-old Caucasian female with predominately hepatic disease that showed resistance to intravenous immunoglobulin (IVIG). The formal diagnosis of KD was made on her 8th day of symptoms. She displayed classical symptoms commencing with fever, followed by peripheral desquamation, strawberry tongue, cervical lymphadenopathy. She became clinically jaundiced with evidence of hepatic artery narrowing on ultrasound that resolved with treatment. Her disease was biphasic and required further IVIG for non-hepatic symptoms. She did not develop coronary aneurysms.

**Conclusion:**

Significant hepatic dysfunction with clinical jaundice is rare in KD without associated gall bladder hydrops and tends to occur in older patients. We describe such a case and review the five described cases in the literature. Diagnostic delay is more common in adolescent patients and given that the prognosis of KD is closely correlated to diagnostic timing and provision of care, it is important to consider Kawasaki Disease in older demographics especially with undiagnosed hepatic disease.

## Background

Kawasaki Disease (KD) is the most common paediatric vasculitis affecting small to medium arteries [1, 2]. Previously referred to as mucocutaneous lymph node syndrome, the majority of morbidity and mortality in KD stems from cardiac involvement with the development of coronary artery aneurysms and arrhythmias [2].

The presentation is typically heralded by a prodromal phase with non-specific viral-like respiratory and gastrointestinal symptoms [1, 2]. Classic findings that form the diagnosis of complete KD as defined by Tomisaku Kawasaki in 1967 are listed in Table 1 [3, 4]. The disease affects between 12 to 215 per 100,000 children under 5 years of age [5–7]. The median age of the disease at diagnosis is 3.4 years and these patients are most likely to present with classical symptoms [4, 8]. Older children and adolescents between 5 and 18 years represent 23% of cases and frequently present with fewer classic features and are less likely to suffer from meningitis, thrombocytosis, and coronary artery aneurysms [7, 9]. They are however more likely to develop hepatitis, cervical lymphadenopathy, and arthralgia [9]. Atypical presentation and reduced awareness of non-paediatric disease mean these patients are more likely to experience diagnostic delay. Consequently 30% of KD patients over the age of 10 years are diagnosed ten days after symptom onset compared to 19% for children under 10 years of age [10].

**Table 1:** Classical clinical features of kawasaki’s disease [3]

A fever lasting ≥5 days with no other clear cause, combined with four or more of the following:	
1. Oral mucosal changes such as injected / fissured lips, pharyngeal injection or strawberry tongue	
2. Bilateral conjunctival injection, without exudate	
3. Polymorphous Rash – Maculopapular, Diffuse Erythroderma, Erythema Multiforme-like	
4. Peripheral changes including erythema or oedema of the palms/soles (in the acute phase), Periungual desquamation (in the subacute phase)	
5. Cervical lymphadenopathy	

KD is typically a self-limiting monophasic disease with prognosis linked to diagnostic timing and subsequent expedient administration of intravenous immunoglobulin (IVIG), within 10 days of symptom onset, shortening the duration of inflammation and preventing organ dysfunction [11]. Treatment resistance has been identified in older demographics with multiple doses of IVIG required to maintain remission. Coupled with the risk of diagnostic delay, these patients subsequently have a worse prognosis [10].

## Case presentation

A previously well 16-year-old female from Central Queensland, Australia presented to a regional hospital with a three-day history of high fevers (39.8 °C) accompanied by myalgia, anorexia and diarrhoea with a single bloody motion on the day of admission. She had not had any sick contacts, recent travel, sexual contact or prior illicit drug use. Her previous history was significant for a hernia repair in 2008, appendectomy in 2011, adenotonsillectomy in 2012, depression, lactose intolerance and autoimmune hypothyroidism. Her immunisations were up-to-date. There were no recent changes to her regular medications with doses of sertraline 25 mg daily and thyroxine 100 micrograms (mcg) during weekdays and 75mcg on weekends. There was a family history of ulcerative colitis and bronchiectasis.

On examination, at initial presentation, she was febrile and appeared unwell. An erythematous maculopapular rash extended over her abdomen and arms; however, no focal signs of infection were apparent.

### Initial investigations

Her laboratory investigations on admission are presented in Table 2.

**Table 2:** Initial investigations upon presentation

Investigation	Result
Haemoglobin	136 g/L (115–160)
White Cell Count	6.1 × 10^9^/L (4–11)
CRP	244 mg/L (< 5)
ESR	45 mm/Hr (< 12)
ALT	239 U/L (< 45)
AST	163 U/L (< 35)
ALP	174 U/L(30–110)
GGT	171 U/L (< 55)
Total Bilirubin	80umol/L (< 20)
Conjugated Bilirubin	58umol/L (< 4)
INR	1.4 (0.9–1.2)
Albumin	18 g/L (29–42)
Fibrinogen	6 g/L (1.7–4.5)
Haptoglobin	2.7 g/L (0.36–1.95)
Blood Film	Normal
Creatinine	63 umol/L (38–82)
Urine - Protein Creatinine Ratio:	58 g/mol creatine (< 15)
Urine – Microscopy, Culture and Sensitivity	No Bacterial Growth
Lipase	83 U/L (< 60)
Creatinine Kinase	94 U/L (28–142)
Thyroid Function Tests	T4 13.0 pmol/L (7–13), TSH 2.9 mU/L(0.3–4.5)
BNP	323 ng/L (< 100)
IgG	5.4 g/L (7–16)

### Clinical Progress

The patient was commenced empirically on cefotaxime 1 g intravenous (IV) 6 hourly and doxycycline 100 mg 12 hourly to cover a potential pulmonary infective source. As noted, she had abnormal liver function tests with a cholestatic predominance. An abdominal ultrasound, performed on Day 4 following symptom onset, demonstrated that her liver measured 15.1 cm in length and had a mildly hyperechoic echotexture. The Gallbladder was reportedly normal with no calculi present. Prominent abdominal lymphadenopathy up to 6 mm was noted.

An infective cause for the fevers was not found despite extensive testing. This included serial blood cultures, serology for Hepatitis A/B/C/E, *Cytomegalovirus* (CMV), Epstein–Barr virus (EBV), Q Fever, Mycoplasma Pneumonia, Leptospirosis, Brucellosis, Anti Streptolysin, Rickettsia, *Human Immunodeficiency Virus*-1 (HIV-1), *Mycobacterium* tuberculosis (TB), *Legionella* pneumophila and Dengue.

Autoimmune studies were negative including Antinuclear Antibodies, antibodies to Extractable Nuclear Antigens, anti-tissue transglutaminase *(anti-tTG*), anti–liver-kidney microsomal antibody-1 (anti-LKM1) and *anti-neutrophil cytoplasmic antibodies* (ANCA).

Subsequently, on day 5 following symptom onset, she developed a diffuse swelling of her hands and feet with an associated progression of her rash that resulted in desquamation of her digits. Her tongue took on a strawberry red appearance, prominent cervical lymphadenopathy developed, her sclera became injected and she became visibly jaundiced.

By day 6 of symptom onset, her serum bilirubin reached its peak of 163umol/L (< 20). The patient then developed an oxygen requirement. Chest x-ray demonstrated mild prominence of pulmonary interstitium suggesting a potential atypical infection. Despite antibiotics she continued to have recurrent febrile episodes with chest imaging, on Day 7 post symptom onset, demonstrating the development of left lower lobe consolidation.

She was given a provisional diagnosis of Kawasaki disease with the differential including an undetected infective aetiology. Assessment for cardiac involvement demonstrated no ECG changes, Troponin I was initially normal on presentation and an echocardiogram was normal for age.

### Treatment

Treatment was commenced on day 8 post symptom onset, with Intravenous Immunoglobulin (IVIG) 2 g/kg total in divided doses, low dose Aspirin 150 mg per day and transfer to a tertiary facility for ongoing specialist management. The patient developed acute pulmonary oedema following her last dose of IVIg which responded to intravenous diuretics. Repeat chest x-ray demonstrated bi-basal opacification that was concerning for an inflammatory process. A CT Pulmonary Angiogram demonstrated bi-basal opacification with a differential diagnosis of infection, pulmonary oedema or atelectasis. There was no evidence of a Pulmonary Embolus.

Repeat ultrasound of her liver demonstrated non-specific hepatic artery tapering to 2.5 mm at the porta hepatis, the Gallbladder had a normal appearance and with no other hepatic or biliary changes. A Renal Artery Duplex ultrasound did not demonstrate renal involvement. Stool *polymerase chain reaction* (PCR) was negative for viral/bacterial/parasitic aetiology though calprotectin was significantly elevated 2200 μg/g (< 50).

Her Troponin I peaked at 0.051μg/L (< 0.04) on Day 8 following symptom onset. A CT coronary angiogram (CTCA) was performed which demonstrated no evidence of stenosis, ectasia or aneurysm. Repeat echocardiography demonstrated a mild pericardial effusion but was otherwise normal.

After 72 h, antibiotics were ceased given marked improvement in chest x-ray finding with diuresis and associated clinical improvement. The patient had a single low-grade fever 2 days after receiving IVIG though continued to improve in all other clinical parameters.

She was subsequently discharged on low-dose aspirin with plans for follow up by multiple specialists.

One week following discharge she was experiencing ongoing low-grade fevers (37.6 °C) and left sided pleuritic chest pain. Over the subsequent days, she became progressively unwell with continued low-grade fevers, malaise, lymphadenopathy and a recurrence of conjunctival injection. She received a further 1 g/kg of IVIg over 2 days. Blood tests at this time demonstrated ESR 80 mm/Hr (1–20), CRP 29 mg/L (0–6), total bilirubin 25umol/L (2–20), normal liver function, normal white cell count though elevated eosinophils 1.53 × 10^9^ (0.04–0.40).

One month later she had made a full clinical and biochemical recovery and aspirin was ceased. Repeat hepatic ultrasound demonstrated resolution of hepatic artery narrowing, a normal appearance of the gall bladder with no intra or extrahepatic bile duct dilation.

## Discussion and conclusions

This case of an adolescent patient presenting with symptoms consistent with KD illustrates prominent hepatic involvement in comparison to the more usual cardiac involvement.

Phenotypic differences between adult and paediatric KD cases are well described, with older patients more likely to have elevated transaminases, cervical lymphadenopathy and arthralgias, although less likely to develop meningitis, thrombocytosis and coronary artery involvement [12]. Core features of KD present in both groups with similar percentages of fevers, pharyngitis, conjunctivitis and desquamating erythematous rashes [12].

Hepatic involvement is a well-documented complication of KD with 65% of adult onset cases developing hepatitis [12–15]. Patients who develop hepatic failure are typically asymptomatic with resolution of liver dysfunction linked to overall disease activity [13]. The pathogenesis of LFT derangement in KD is incompletely understood but is thought to be multifactorial [13]. This patients disease was not monophasic which has previously been described in older patients and particularly those with hepatic involvement [7].

In a subset of cases, patients present with obstructive jaundice and hydrops of the gall bladder. This has been theorised to be due to compressive lymphadenopathy of the porta hepatis [16]. Less commonly, patients with KD have been found to present with clinical jaundice but without sonographic evidence of hydrops or mechanical obstruction [17–21].

To our knowledge there have been five previous reported cases of obstructive jaundice without evidence of hydrops of the gallbladder which we have summarised in Table 3 [17–21]. In these cases, patients demonstrated normal hepatobiliary anatomy on standard imaging modalities with three reports of nonspecific gallbladder/biliary dilatation [17–21]. In all cases LFTs returned to normal ranges following administration of IVIG although long term maintenance of normal liver function was not recorded [17–21]. In three cases, a liver biopsy had been performed which demonstrated evidence of intrahepatic cholangitis and bile duct epithelium necrosis without lobular inflammatory infiltrate, or liver cell necrosis that would be found in a viral aetiology. While this histological picture is potentially in keeping with ischemia, no definite vasculitic changes were found, although this may have been due to a lack of sufficiently sized blood vessels in the sample [17]. This suggests that while obstructive jaundice associated with hydrops of the gallbladder may in part be related to localised lymphadenopathy causing physical obstruction, vasculitis involving the liver remains a possibility.

**Table 3:** Clinical Features of patients with Kawasaki Disease presenting with obstructive jaundice

Reference	Age	Hepato-megaly	Icterus	Abdominal Pain	Serum ALT(x ULN)	Serum AST(x ULN)	Serum ALP(x ULN)	Serum GGT(x ULN)	Serum Bilirubin(umol/L)	Pulmo-nary Involvement	Treatment
Our patient	16	+	+	+	5.3	4.66	1.58	3.1	163	+	IVIG 2 g/kg in divided doses of 0.8 g/kg on the first day and 1.2 g/kg on the second day. Oral Aspirin. Refractory Disease requiring a further 1 g/kg of IVIG over 2 days.
Bader-Meunier et al. [17]	3.5	+	+	NR	5	2.5	4	15	23.9	–	IVIG 1 g/kg/day. Not refractory
Keeling et al. [18]	12	+	+	+	1.91	0.80	1.43	2.63	48.9	–	IVIG 1.6 g/kg/day. Oral Aspirin. Not refractory
McMahon et al. [19]	20	NR	+	+	4.74	2.28	1.83	4.45	134.4	–	IVIG 2 g/kg/day. Oral Aspirin. Not Refractory.
Valentini et al. [20]	6	NR	+	+	9.96	11.87	1	10.88	121.4	–	IVIG 2 g/kg/day. Oral Aspirin. Not Refractory.
Vázquez et al. [21]	6	+	+	NR	NR	NR	3.21	4.02	98.3	–	IVIG 2 g/kg/day. Refractory Disease requiring further IVIG 72 h after the 1st dose and 48 h of Methylprednisolone 15-30 mg/kg for 2 days. Oral Aspirin.

*NR* Not recorded, *ULN* Upper Limit of Normal

In our case, there was no hydrops of the gallbladder found; however, the non-specific tapering of the hepatic artery seen on ultrasound that resolved with treatment potentially represent vasculitis. The patients identified in the available literature including our patient were older than the median diagnostic age for KD. In infancy an initial broad tolerance prevents reactions against maternal antigens though as the immune system matures autoimmunity becomes more likely [22]. This affects both the innate and adaptive immune system [22]. With increasing expression of toll-like receptors in the innate immune system and increasing selectivity of B and T cell response [22]. Therefore it is speculated that additionally organ systems may be involved in older children and adults as their immune system incorrectly identifies additional antigens [22].

This case was further complicated through the patient’s development of basal consolidation that potentially represented infection, atelectasis, oedema or an inflammatory process. Pulmonary changes responded promptly to the combination of intravenous antibiotics, diuresis and IVIG. Further delineation of the aetiology was not possible however pulmonary involvement of KD is recognised but is rare with a reported incidence on 1.3% [23]. In previous published cases the diagnosis is considered when antimicrobials are not effective leading to diagnostic delay and subsequent association with the development of coronary artery aneurysms [23, 24]. Compared to the previous cases of KD with Cholestatic LFT derangement this is the only case to demonstrate potential pulmonary involvement.

We have reported a rare case of adolescent onset KD presenting with cholestatic jaundice with a relapsing course which required a second administration of intravenous immunoglobulin. Despite this she made a complete clinical recovery receiving appropriate treatment within 10 days following initial diagnostic uncertainty. However, the optimal time period for IVIG administration in this particular population group is unclear. As such it is uncertain if the time in which the patient was undiagnosed contributed to the need for further therapy or whether this population group experiences a longer and less monophasic disease.

In summary, hepatic predominant presentations should be considered as an uncommon variant of KD. This presentation appears to be more common in older age groups where treatment resistance is also more common. Subsequently hepatic involvement should suggest the need for closer monitoring in the convalescent phase to exclude recurrence. The mechanism for this presentation is unclear and further research is required. Fortunately, in most cases this is a transient phenomenon, negating the need for invasive investigation.

## Data Availability

Data for this study is stored on Queensland Health pathology database. Access to this data is restricted to Queensland Health employees with a valid clinical reason for access. All data generated or analysed during this study are included in this published article.

## Abbreviations

ALP: Alkaline phosphatase
ALT: Alanine transaminase
ANCA: Anti-neutrophil cytoplasmic antibodies
anti-LKM1: Anti–liver-kidney microsomal antibody-1
anti-tTG: Anti-tissue transglutaminase
AST: Aspartate aminotransferase
ESR: Erythrocyte sedimentation rate
CRP: C reactive Protein
GGT: Gamma-glutamyltransferase
IVIG: Intravenous Immunoglobulin
KD: Kawasaki Disease
LFT: Liver Function Test
NR: Not Recorded
ULN: Upper Limit of Normal
